# Green alternative for sodium metabisulfite substitution: Comparison of bacterial and fungal proteases effect in hard biscuit making

**DOI:** 10.1177/10820132231152279

**Published:** 2023-01-22

**Authors:** Farzad Saberi, Fatemeh Kouhsari, Nicola Gasparre

**Affiliations:** 1 Department of Food Science and Technology, Science and Research Branch, Islamic Azad University, Tehran, Iran; 2 Department of Research and Development, Zarkam Company, Zar Industrial and Research Group, Hashtgerd, Iran; 3 Department of Food Science, Engineering and Technology, College of Agriculture and Natural Resources, University of Tehran, Karaj, Iran; 4 Department of Food and Human Nutritional Sciences, 8664 University of Manitoba, Winnipeg, Canada

**Keywords:** hard biscuits, enzymes, bacterial protease, fungal protease, structure

## Abstract

Sodium metabisulfite is one of the most employed reducing agents in hard biscuit making. The recent results about its adverse effects on human health have pushed us to look in new safer and greener directions. Two different proteases, from distinct strains (bacterial and fungal), were selected and their effects on the dough thermomechanical performances, texture, and structure of the hard biscuits were compared with those obtained from the sodium metabisulfite. Doughs treated with fungal protease showed higher stability during mixing and higher consistency throughout the heating stage. On the other hand, bacterial protease had the greatest weakening effect on protein with a marked reduction of starch gelatinization. Doughs processed with fungal enzymes reached similar values to those containing sodium metabisulfite. In terms of hardness, no significant (*p* < 0.05) differences were found between biscuits made with bacterial protease and sodium metabisulfite. Analysis of the volumetric characteristics highlighted that bacterial enzymes gave higher values of specific volume and surface area. Regarding the structure of the hard biscuits, sodium metabisulfite produced a more uniform structure with fewer and smaller pockets as compared with the samples treated with proteases.

## INTRODUCTION

Biscuits belong to the vast category of cereal-based products, and they are particularly appreciated for their highly crispy and flat texture as well as for their sweet taste. Hard biscuits are produced by a rotary cutter system that allows obtaining adequate dough stretching and texture before shaping (Manley, 2011). One of the most important features of a biscuit is its texture which depends on many factors such as the flour protein content, the characteristics of the fat utilized, and the type of baking oven employed that regulates the heat transfer mechanism (Chevallier et al., 2000).

Regarding the protein content of the flour, gluten plays a major role in terms of rheology and thermomechanical properties of the dough. Owing to the high elasticity and strength that gluten gives to the dough system, the final shapes, and sizes of the hard biscuits are dramatically affected, resulting in different from each other. For this reason, in the recent years, the food industry has mostly focused on this group of storage protein to shape the final dough characteristics.

Due to its action on the protein network, sodium metabisulfite (SMBS) represents one of the most used process aiders in crackers and hard biscuits manufacturing. Through the cleaving of the disulfide bonds within or between proteins, this reducing agent decreases the dough strength and improves the system extensibility for better sheeting (Pedersen et al., 2005). Doughs treated with SMBS require less mixing time and lamination may not be needed. Nevertheless, these benefits are complemented by some downsides such as lighter color and unwanted aftertaste (Ahmad et al., 2021). In addition, due to the adverse reactions related to the consumption of sulfites, the use of SMBS has been reduced in many countries (Vally and Misso, 2012). To eliminate these drawbacks the modern food industry has set out to search for other more sustainable and safer alternatives with the aim to replace SMBS.

Among the possible SMBS substitution strategies, the use of proteolytic enzymes (proteases) could represent a valid way to take them. In fact, thanks to their high substrate specificity, enzymes find wide applicability in the food technology field, improving quality and process efficiency (Akbari et al., 2021). The performance of the proteases, especially in breadmaking, has been largely described; they are mainly responsible for a reduction of the dough tenacity and for an increase in the loaf-specific volume (Caballero et al., 2007). To date, few studies have been carried out about the use of proteases in biscuit formulations. Kara et al. (2005) found that proteases from bacterial sources had a greater effect on the gliadin, while plant proteases impacted the glutenin functionality. In addition, when proteases were used as an SMBS replacer, flavor and color improved during baking. Pedersen et al. (2005) described the effect of SMBS and proteases on biscuits prepared with wheat flour from different cultivars. Creep recovery and oscillation test results showed an increase in the extensibility and a reduction in the elasticity of the doughs containing SMBS and proteolytic enzymes culminating in a deep modification of the dimensional properties of the biscuits. According to the results of the dynamic oscillatory measurements carried out by Oliver et al. (1996), doughs prepared with bacterial protease showed higher elastic and viscous moduli than those found in the samples containing SMBS.

As of right now, a complete study that would include an evaluation of the dough mechanical properties, structure and texture of the hard biscuits would be required for a better understanding of the changes brought by SMBS and proteases addition in biscuit making. Due to this, the aim of this study was to evaluate the potential use of proteolytic enzymes as a substitute for SMBS. To reach this goal, samples were prepared using two different proteases from bacterial and fungal sources. Their performances on the thermomechanical behavior of the doughs were assessed and compared with those led by the SMBS. Then, confocal laser scanning microscopy (CLSM) and scanning electron microscopy (SEM) were employed to explore the different interactions among the dough components and the internal microstructure of the final products, respectively. An evaluation of the texture properties of the final samples allowed us to get a full picture of the proteases’ role as SMBS substitute in hard biscuit making.

## MATERIALS AND METHODS

Regarding the biscuit preparation, wheat flour and wheat starch were purchased from Zar industrial group (Hashtgerd, Iran). The remainder of the materials: icing sugar from Debel (Khazaee, Khuzestan province, Iran), table salt (150–250 µm) was provided by Taban Co. (Tehran, Iran), sodium metabisulfite (assay: 99.1%, SO_2_ content: 66.02%), sodium acid pyrophosphate 28 (SAPP28, assay: 96%, P_2_O_5_: 64%) and sodium bicarbonate (NaHCO_3_ content: 99.5%, pH value: 8.2) were purchased from Fooding Co. (Shanghai, China), Glucose syrup (DE = 42, brix: 82, Zar fructose Co., Hashtgerd, Iran), soy lecithin (acetone insoluble %: 63, acid insoluble (mg KOH/g): 29.5, effective HLB value: 4, Adlec, ADM wild, USA), bacterial protease from *Bacillus amyloliquefacians* (Bakezyme B500 BG, enzymatic activity: 90000 PC/g ± 5%) and fungal protease from *Aspergillus oryzae* (Bakezyme PPU 95000, enzymatic activity: 95000 PC/g ± 5%) were purchased from DSM (Delft, Netherlands), butter flavor from Firmenich (Geneva, Switzerland) and shortening (slip melting point: 41.1°C, biscuitine 500) from Loders Croklaan (Pasir Gudang, Johor Malaysia). All other reagents were of analytical grade.

### Crude protein, gluten content and gluten index of flour

Following the approved methods of analysis from AACC (2000), protein content (46-11A), as well as wet and dry gluten content (38-10.01) were determined. Gluten index was measured using a gluten index analyzer (2100, Bastak, Turkey) according to the standard method 158 of ICC (1995).

### Thermomechanical behavior of the doughs

The thermomechanical behavior of the doughs, prepared with flour, water and treatment agents was evaluated through the Mixolab device (CHOPIN Technologies, Cedex, France). SMBS was added following the concentration (0.036 mg/100 g of flour) proposed by Pedersen et al. (2005), while both enzyme additions were based on the levels (0.0033 mg/100 g of flour) recommended by the suppliers. The preparation and characterization of all the doughs were in accordance with the standard method (54–60.01) of the AACC (2000) with slight modifications in which SMBS and each enzyme were premixed with the flour before adding to the Mixolab bowl. The moisture content of the flour was measured using an NIT device (Infracont Mininfra Scan-T Plus, Ankara, Turkey). Dough hydration was obtained through the Mixolab device by adjusting water until reach the optimum dough consistency at 1.1 Nm. The parameters obtained from the recorded curve were: water absorption (amount of water required for the dough to reach a torque of 1.1 Nm), maximum torque reached during mixing which corresponded to the C1 point, C2 or protein weakening as a function of mechanical work and temperature, C3 was related with the starch gelatinization, C4 point relative to the lowest consistency reached during the heating stage and C5 referred to the starch retrogradation during the cooling stage. This test was carried out in triplicate for all the samples.

### Free sulfhydryl groups determination

The free sulfhydryl groups (-SH) content of the samples was determined using the Ellman's coloration method with slight modifications (Beveridge et al., 1974). The determination was carried out during the mixing stage (every minute) and during the rest period (every 10 min). Each dough was freeze-dried in a freeze-drier (Christ Alpha 1-2 LDplus Freeze Dryer, −55 °C ice condenser, Shropshire, UK) and powdered. A total of 50 mg of the lyophilized dough powder was dispersed in 1.0 mL Tris-Gly buffer (pH 8.0, containing of 8 M urea and 3 mM 5, 5′-Dithiobis-2-nitrobenzoic acid). After that, 4.70 g of guanidine hydrochloride was added to the prepared mixture and diluted with 10 mL of buffer. To calculate the free -SH, 1 mL of the sample solution was mixed with 0.05 mL of the Ellman's reagent and 4 mL of the urea-guanidine hydrochloride (containing 8 M urea and 5 M guanidine hydrochloride). The mixture was let to react at 25 °C for 20 min in darkness and the absorbance was read at 412 nm using a spectrophotometer (AA320N, Wincom laboratory equipments, Shanghai, China). -SH content was calculated through the following equation (Zhao et al., 2013):

(1) $$\mathrm{Free}\;\mathrm{SH}\;(\mu \mathrm{mol}/g)=73.53\times A_{412}\times \frac{D}{C}$$

where $A_{412}$ represents the absorbance at 412 nm; C represents the sample concentration in mg/ml and D represents the dilution factor which is 5.02.

### Biscuit-making process

Hard biscuits were prepared according to the following formulation: wheat flour 56.815 g/100 g, vegetable fat 8 g/100 g, icing sugar 12.5 g/100 g, water 16 g/100 g, protease enzyme 0.035 g/100 g) or SMBS: 0.036 g/100 g, sodium bicarbonate 0.3 g/100 g, sodium acid pyrophosphate 0.3 g/100 g, lecithin 0.1 g/100 g, native wheat starch 4 g/100 g, glucose syrup: 1.5 g/100 g, salt 0.3 g/100 g and butter flavor 0.15 g/100 g. Initially, the temperature of the double Z mixer (LKII 1, Hermann linden, Marienheide, Germany) was set to 35°C and then vegetable fat, lecithin, sodium bicarbonate, sodium acid pyrophosphate, and flavoring were mixed for 3 min at 50/min. Then, water, glucose syrup and table salt were added and mixed for another 2 min. Finally, wheat flour with SMBS or enzymes was added and mixed for another 5 min until obtaining homogeneous and stretchable dough. Then, each one of the prepared doughs was covered by a plastic film and left to rest for 30 min. After that, a dough sheet was prepared via a Rondo dough-sheeter (Econom model, Burgdorf, Switzerland) according to the following procedure: dough was sheeted until get 25 mm of thickness and then, it was manually placed on the dough-sheeter belt and subsequently sheeted again to obtain a thickness of 20 mm. Then, the sheet became thinner by passing it through a 15 mm gap. This procedure was repeated three times through a 10, 6 and 3 mm gaps. Finally, the dough sheet was cut into 4 × 10 mm pieces and put on a tray and baked in an electrical oven (D-2, Sveba dahlen, Fristad, Sweden) for 10 min at 200 °C.

### Confocal laser scanning microscopy (CLSM) of the doughs

Following the method of Reynaud et al. (2020) with some modifications, the prepared doughs were separately stained with Nile Red (Sigma Aldrich, MA, USA) and Alexa Fluor 546 (Biocompare, San Francisco, USA) in order to visualize fat and protein, respectively. A solution containing 0.5 mg/mL of Nile Red and 0.17 mg/mL of Alexa Fluor 546 was prepared using dimethylformamide as solvent. The fluorophores were kept and protected from light at −20 °C to avoid any possible degradation. Eight dough samples were taken from each production. Samples (slice of 10 µm of thickness) were cut off in the cryostat microtome chamber (HM 500 OM, Microm International, USA) set at −25 °C. Before the slide cover was placed on the top, dough slices were covered with 15 µL of fluorophore solution. Mounted slides were stored for 24 h before observation to ensure a complete diffusion of the fluorophores. For excitation purposes, a Nikon CLSM (Model A1) with a green laser at 561 nm was used. The fluorescence emission of the two dyes was detected between 563 nm and 643 nm and spectral de-convolution was performed.

### Texture profile analysis of the doughs

Texture profile analysis (TPA) of the doughs was evaluated in terms of hardness, springiness, adhesiveness and cohesiveness using a texture analyzer (TA.XT plus 100C, Stable Microsystems, Surrey, England) according to Krystyjan et al. (2015) with slight modifications. The dough pot was filled with freshly prepared dough. The aeration plunger squeezed the dough to extract the air pockets. After removing the plunger, the flattening plunger was inserted into the dough pot and compressed until obtaining a leveled surface. The test was performed using a 5 kg load cell, a 6-mm cylindrical stainless-steel probe positioned at 20 mm above the dough pot surface. The texture analyzer settings were test speed of 1 mm/s, penetration depth of 15 mm and a trigger force was 5 g, total test time was 30 s. Thirteen replicates were run for each sample.

### Characterization of biscuits

Biscuits were characterized assessing texture, volume, and microstructure. Three-point bend test was carried out using the three-point bending rig (HDP/3 PB, Stable Microsystems, Surrey, England). The span between the supports was 40 mm. Pre-test speed, test speed, post-test speed and distance were 1.0 mm/s, 2.0 mm/s, 10.0 mm/s and 6.0 mm, respectively. A 5 kg load cell was used, and the data acquisition rate was set at 500 pps. The measured parameters were hardness and fracturability. The test was performed in triplicate for each sample.

The volume of the biscuits was evaluated using a 3D laser-based scanner (Volscan profiler C, Stable microsystems, Surrey, England). Settings for the measurement were: vertical speed and rotation speed were set at fine but slow and 0.5 rps, respectively. To record the biscuits’ volume during baking, the method described by Saberi et al. (2021) was used with some modifications. Briefly, the biscuits were collected at the end of the baking and were positioned in the scanner, their density, specific volume and surface area were calculated according to the following equations:

(2) V=l×w×h

where *V*, *l*, *w* and *h* are volume, length, width and height of biscuit, respectively.

(3) $$\upsilon =\frac{V}{m}$$

where ʋ, *V* and m are specific volume, volume and mass of the biscuit, respectively.

### Microstructural analysis of the biscuits

After baking, biscuits were put into a zip plastic bag, sealed and stored at −18 °C for 90 min. Then, they were manually broken and analyzed using a scanning electron microscope (VEGA3, Tescan, Kohoutovice, Czech Republic) using a voltage of 20 KV and 50× magnification. To quantitatively characterize the surface microstructure of the biscuit samples, image analysis was applied to the SEM using MATLAB R2018b software (Mathworks Inc., Natick, MA, USA). Each image was converted from RGB format to eight bits (grayscale) using the software. Then, the contrast of the images was enhanced, and they were divided using the Otsu algorithm, which provides highly uniform binary images (Saberi et al., 2021). Porosity was measured as the ratio of the sum of the porous area and the whole area. The surface characteristics include energy, contrast, correlation, homogeneity and entropy that were calculated from the gray-level co-occurrence matrixes of each sample image. Features of image texture were calculated based on the following equations (Saberi et al., 2019):

(4) $$Energy=\sqrt{\underset{i}{\sum }\;\underset{j}{\sum }\;p_{d,\theta }^{2}(i,\;j)}$$

(5) $$Contrast=\;\overset{d}{\underset{n=0}{\sum }}\;n^{2}\left(\underset{i}{\sum }\;\underset{|i-j|=n}{\underset{i}{\sum }\;p_{d,\theta }(i,j)}\right)$$

(6) $$Homogenity=\;\underset{i,j}{\sum }\frac{\;p_{d,\theta }\;(i,\;j)}{1+|i-j|}$$

(7) $$Entropy=\;-\underset{i}{\sum }\;\underset{j}{\sum }\;p_{d,\theta }(i,\;j)\log\;(\;p_{d,\theta }(i,\;j))$$

where $p_{d,\theta }(i,j)\;$ counts the number of neighboring pair pixels with gray values of *i* and *j* at a particular distance of *d* and *a* given the direction of *θ*. *µ* and *σ* are mean and standard deviation of $p_{d,\theta }(i,j)$ , respectively.

### Surface color

The surface color of the biscuits was measured by the Ultra Scan PRO colorimeter (Hunter Associates Laboratory, Inc., Reston, VA, USA). The measured parameters were L* [lightness: 0 = black, 100 = white], a* [greenness (−), redness ( + )], and b* [blueness (−), yellowness ( + )]. Three samples from each batch were measured at the same point (Saberi et al., 2019). The total difference in color (ΔE) among the enzyme-treated biscuits was measured using the following equation:

(8) $$\Delta E=[{\left(\Delta L\right)}^{2\;}+{\left(\Delta a\right)}^{2}+{\left(\Delta b\right)}^{2\;}{]}^{\frac{1}{2}}$$

Only values of ΔE ≥ 3 mean that differences are visible to the human eye. Also, Browning Index (BI) was calculated using the following equation (Ureta et al., 2014):

(9) $$\mathrm{BI}\;=\;\frac{\left[100\left(\frac{a+1.75L}{5.645L\;+a-3.012b}-0.31\right)\right]}{0.17}$$

### Statistical analysis

To run the analysis of variance (ANOVA) and the significance mean levels (Duncan) (*p* < 0.05), all the data obtained were processed through the SAS software version 9.2 (SAS Institute, Cary, NC). Minitab 18 software (Minitab, LLC, Penn State University) was used to build the principal component analysis (PCA).

## RESULTS AND DISCUSSION

### Thermomechanical behavior of the doughs

**Figure 1.**
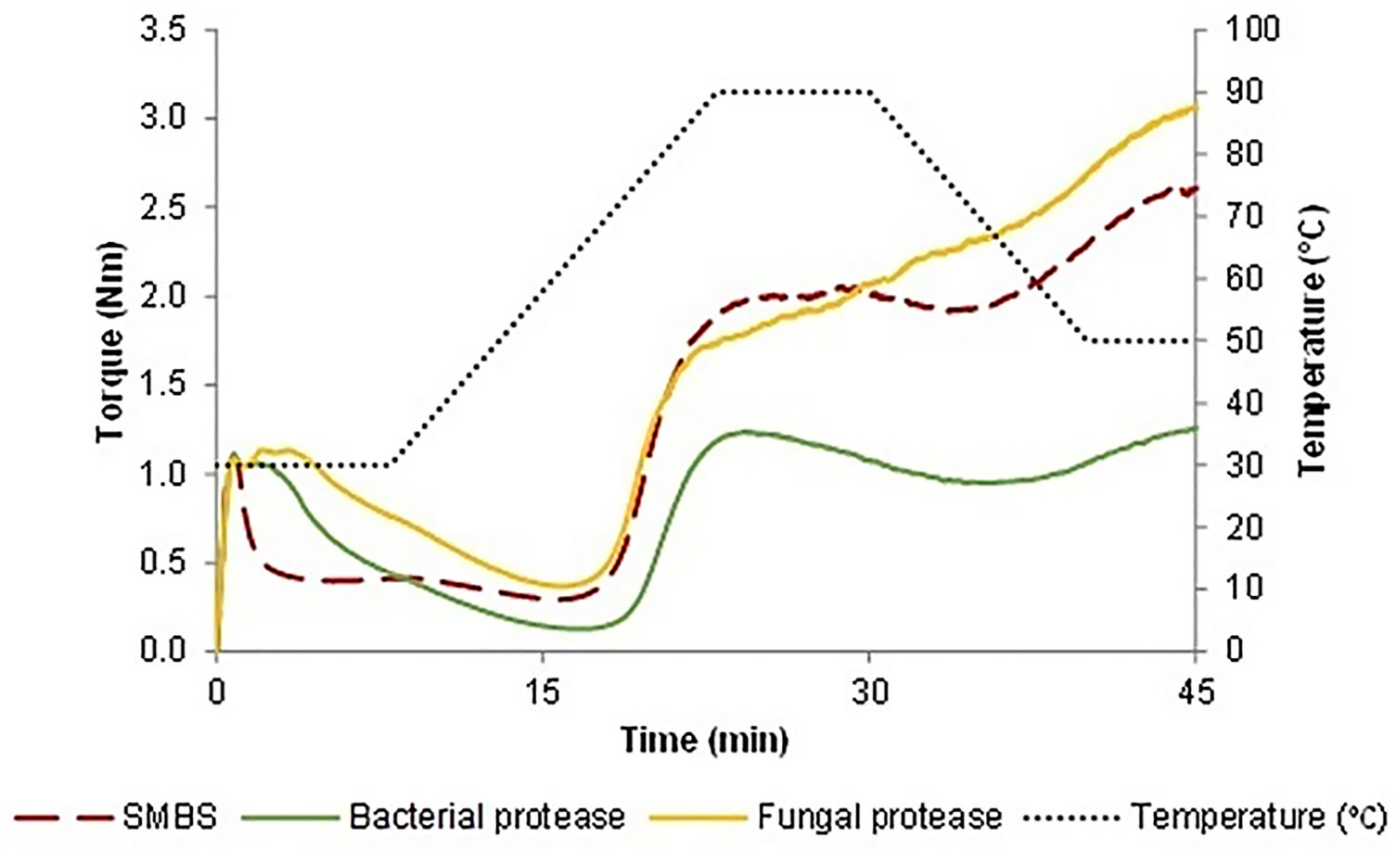
Mixolab^®^ plot representing the thermomechanical behavior of the SMBS and proteases treated doughs.

**Figure 2.**
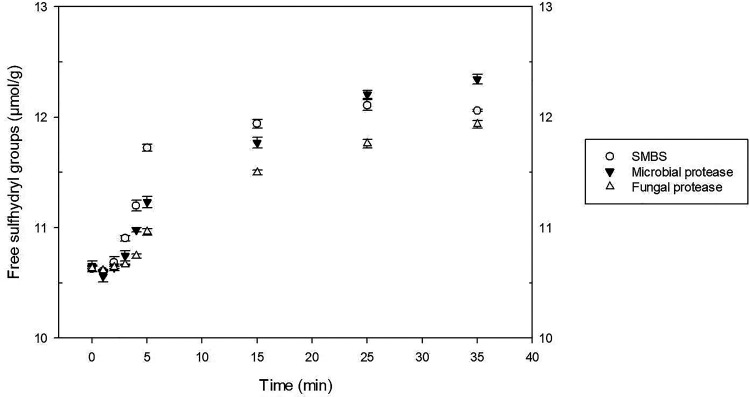
Free sulfhydryl groups of flours treated with sodium metabisulfate and proteases during mixing (5 min) and resting time (30 min).

Moisture content, protein, wet gluten and gluten index of the flour were 13.51, 12.07, 26.2, and 86 g/100 g, respectively. The thermomechanical behavior of the doughs, while subjected to a dual mixing and to different cycles of heating and cooling, was evaluated using the Mixolab device (Gasparre and Rosell, 2019). The water absorption required to get a torque of 1.1 Nm corresponded to 52.24 g/100 g for all the samples. The plots confirmed the impact of the additive and processing aids on doughs along with mixing-heating-cooling (Figure 1). Briefly, the first zone is about the dough development, here the stability during mixing can be valued until the onset torque reduction caused by the overmixing which corresponds with the protein weakening. Then, as the temperature grew, consistency continued to decrease followed by a slight increase related to the starch gelatinization. After a small torque reduction, more precisely during the cooling stage, the device registered a final rise of the linear force mainly brought on by the starch retrogradation (Rosell et al., 2007). Results reported in Table 1 allow us to compare the performances of SMBS and enzymes. Regarding the stability during mixing that is strongly related to the formation of disulfide bridges, samples containing SMBS had the lowest value (0.93 min) followed by the doughs treated with bacterial (2.70 min) and fungal protease (4.50 min). The lowest stability caused by the SMBS is the result of a missing disulfide bond creation. In fact, in the water, this reducing agent is hydrolyzed into bisulfite that reacts with the protein disulfide groups and generates free sulfhydryl groups and a sulfate ion, blocking the new formation of the disulfide bonds (Singh et al., 1990). The determination of the -SH (Figure 2) during mixing showed that the highest quantity was present in the dough containing SMBS confirming its reducing action. As pointed out before, gluten strength comes down to the interactions, principally through disulfide bridges, between gliadin and glutenin that define the extensibility and elasticity of the dough. Proteases, that exhibit ample substrate specificity for gluten proteins, hydrolyze the peptide bonds involved in the hydrophobic interactions, decreasing the final protein molecular weight (Kong et al., 2007). The chain length reduction operated by the proteases finds expression in a dough consistency drops. Bacterial protease provoked the strongest protein weakening followed by the SMBS, while the fungal strain slightly affected this parameter (Table 1). Similar trend was observed by other authors in which the wheat dough consistency decreasing was directly proportional to the level of the added proteases (Kara et al., 2005; Ekramian et al., 2021). The maximum consistency registered during the heating stage, which is largely affected by the starch gelatinization, ranged from 1.24 to 2.05 Nm. The highest value was reached by the SMBS control, while the bacterial protease had the greatest impact in terms of consistency reduction. The lower torque values found in the enzymes-treated samples could be due to a solubility change in the wheat gluten. In fact, the increased hydrophilic nature of the smaller protein chains generated by the hydrolysis of the enzymes may have provoked competition against starch for the water (Kong et al., 2007). The limited water available for the starch swelling could be the cause of the observed consistency decrease during heating. Regarding the minimum consistency registered during the heating stage (C4), samples treated with fungal protease or SMBS showed the highest results as opposed to the bacterial protease that induced again the most pronounced effect. The slight toque reduction carried out by the fungal protease could be explained by its thermolability. In fact, it was found that these proteases would be deactivated quickly at temperatures around 65 °C, while those from bacterial strain continued also during heating, resulting to be more thermostable. Similarly, dough treated with bacterial protease exhibited the lowest consistency (1.25 Nm) during the cooling stage while samples with fungal protease and SMBS reached 3.05 and 2.84 Nm, respectively.

**Table 1.** Thermomechanical behavior of the doughs.

Sample	Mixing consistency (Nm)	Mixing stability (min)	Protein weakening (Nm)	Starch gelatinization (Nm)	Heating consistency (Nm)	Starch gelling (Nm)
SMBS	1.09 ± 0.02^ a^	0.93 ± 0.09^ b^	0.27 ± 0.02^ b^	2.05 ± 0.14^ a^	1.92 ± 0.12^ a^	2.61 ± 0.14^ b^
Bacterial protease	1.11 ± 0.01^ a^	2.70 ± 0.08^ c^	0.13 ± 0.01^ c^	1.24 ± 0.09^ c^	0.95 ± 0.10^ b^	1.25 ± 0.09^ c^
Fungal protease	1.13 ± 0.02^ a^	4.50 ± 0.12^ a^	0.35 ± 0.01^ a^	1.74 ± 0.10^ b^	2.06 ± 0.13^ a^	3.05 ± 0.12^ a^

Different letters within the same parameter differ significantly (*p* < 0.05).

### CLSM analysis

**Figure 3.**
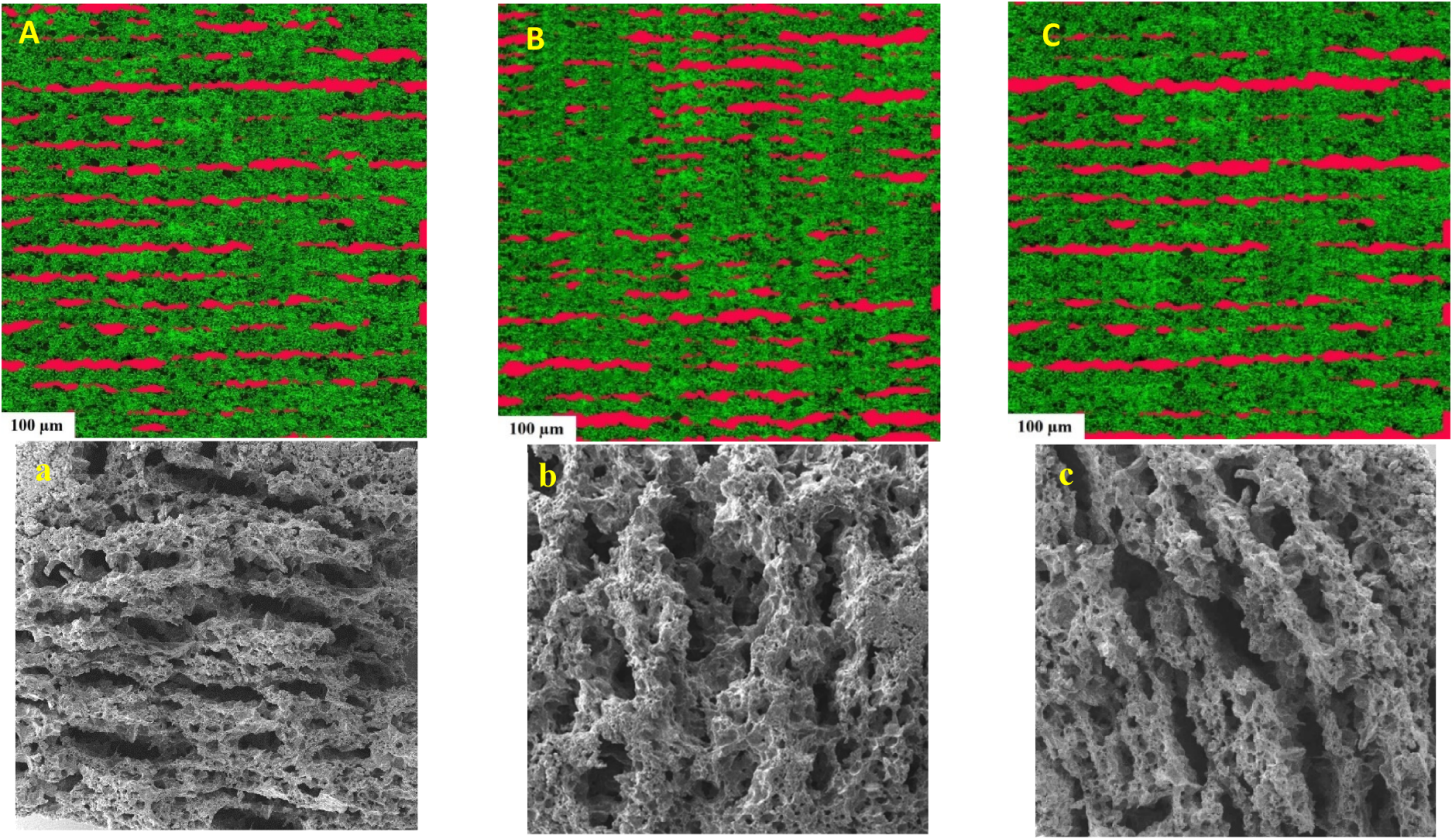
Confocal laser scanning microscopy images of the samples treated with sodium metabisulfite (A), bacterial protease (B), fungal protease (C). SEM images of the samples treated with sodium metabisulfite (a), bacterial proteases (b), fungal protease (c) at 50× magnification.

Confocal laser scanning microscopy (CLSM) was applied to identify the distribution of the components within the dough microstructure. Nile Red was used to stain all hydrophobic compounds (fat layers) visible in red color while Alexa Fluor 546 turns to green the proteinaceous matrix (gluten). Figure 3 (A, B and C) displays SMBS, bacterial and fungal proteases-treated doughs, respectively. Regarding the SMBS sample (3A), fat particles (red areas) appeared to be longer and thinner and better distributed in the protein matrix (green areas) of the biscuit. It is known that liquid oil naturally tends to phase separately, as confirmed by the thick elongated red zones. This phenomenon becomes more evident when the dough texture is elastic and hard enough, impeding an easy distribution of the oil particles. Dough compactness works as a barrier for the oil droplets making their spreading harder through the dough matrix (Jacob and Leelavathi, 2007). On the other hand, image 3B related to the dough treated with the bacterial protease presented bigger hydrophobic red areas more evenly spread compared to those of the other protease sample. Thus, the action of the bacterial proteases led to a broken and weaker gluten network where oil droplets were evenly dispersed (Dreher et al., 2020). In the case of the fungal proteases-treated biscuits (Figure 3C), the presence of the hydrophobic layers seemed to be lower than that observed in Figure 3B, but they resulted to be longer and thicker.

### TPA of the doughs

Results coming from the TPA analysis of the doughs are summarized in Table 2. The values of the hardness ranged from 169 (bacterial protease) to 211 g (fungal protease) with SMBS dough (190 g) that resulted softer. Springiness that refers to the capacity to recuperate the initial shape after a deformation resulted significantly (*p* < 0.05) lower in the samples made with fungal protease, while no significant differences were observed in the samples treated with the bacterial enzymes and SMBS. Dough cohesiveness increased with the proteins breakage, likely due to the higher number of smaller protein chains with a higher capacity to plasticize and bond other ingredients able to form a continuous phase of the dough (Curti et al., 2014). In this regard, both enzymes were significantly (*p* < 0.05) different from each other, with the bacterial ones that showed the highest values (0.46); however, no significant (*p* < 0.05) differences were found among all the samples. To measure how sticky the dough was, adhesiveness was considered and the bacterial enzymes caused the greatest effect (Table 2). This increase in stickiness could be due to a higher starch release caused by a weaker gluten network. In fact, bacterial-treated dough showed the lowest value of protein weakening obtained during the evaluation of the thermomechanical properties.

**Table 2.** Texture characteristics of doughs and biscuits. Volumetric analysis, color and microstructural features of biscuits.

		Parameters	SMBS	Bacterial protease	Fungal protease
Texture analysis	Dough	Hardness (g)	190 ± 12 ^b^	169 ± 1° ^c^	211 ± 12^ a^
		Springiness	0.96 ± 0.01 ^b^	0.93 ± 0.01^ c^	0.98 ± 0.01 ^ab^
		Cohesiveness	0.42 ± 0.02 ^ab^	0.46 ± 0.02 ^a^	0.39 ± 0.02 ^b^
		Adhesiveness (g·s)	2.0 ± 0.1 ^b^	2.5 ± 0. 2 ^a^	2.0 ± 0.2^ b^
	Biscuit	Fracturability (g)	2667 ± 17 ^a^	2419 ± 33 ^b^	2368 ± 26 ^b^
		Hardness (g)	6008 ± 43 ^b^	5957 ± 80 ^b^	6287 ± 76 ^a^
Volumetric analysis		Density (g/cm^3^)	1.24 ± 0.02 ^ab^	1.20 ± 0.03^ b^	1.27 ± 0.03 ^a^
		Specific volume (ml/g)	1.60 ± 0.01 ^b^	1.64 ± 0.02^ a^	1.57 ± 0.02 ^b^
		Surface area (mm^2^)	17522 ± 46 ^b^	18155 ± 82 ^a^	17152 ± 75 ^c^
Color		L*	75.09 ± 0.75 ^a^	62.18 ± 0.8 ^c^	69.34 ± 0.76 ^b^
		a*	2.64 ± 0.14^c^	4.64 ± 0.2 ^a^	3.62 ± 0.1 ^b^
		b*	11.37 ± 0.31 ^c^	16.41 ± 0.15 ^a^	13.04 ± 0.21 ^b^
		BI	19.79 ± 0.29^ c^	35.57 ± 0.52 ^a^	24.26 ± 0.43 ^b^
		ΔE	6.16 ± 0.29^ c^	19.75 ± 0.37 ^a^	11.79 ± 0.22 ^b^
Image processing parameters		Porosity	0.648 ± 0.015 ^b^	0.679 ± 0.017^ a^	0.622 ± 0.014 ^c^
		Energy	0.00028 ± 0.00003 ^a^	0.00023 ± 0.00004^ b^	0.00031 ± 0.00003^ a^
		Contrast	1743 ± 32 ^b^	1854 ± 47^ a^	1442 ± 41 ^c^
		Homogeneity	0.17 ± 0.01 ^a^	0.11 ± 0.01^ b^	0.14 ± 0.02^ a^
		Entropy	14.29 ± 0.3 ^b^	19.13 ± 0.6^ a^	11.74 ± 0.4^ c^

Different letters within the same parameter differ significantly (*p* < 0.05).

### Texture analysis of the biscuits

Fracturability and hardness were the parameters evaluated to better describe the texture attributes of the biscuits (Table 2). Regarding the fracturability, no significant difference (*p* > 0.05) between both sets of enzymatically treated biscuits was observed, nevertheless, they were significantly (*P* < 0.05) lower than those with SMBS. In terms of hardness, fungal proteases produced the biscuits with the lowest values (6287 g), while the bacterial strain proteases contribute to creating a final sample with stiffness (5957 g) similar to that observed in the SMBS control (6008 g). Precisely about the last result, the same behavior was described by Oliver et al. (1996), semi-sweet biscuits made by SMBS, and bacterial enzymes presented no significant (*p* < 0.05) difference in terms of hardness.

### Volumetric analysis

Density, specific volume and surface area of biscuits belong to the volumetric analysis and are reported in Table 2. Based on the obtained findings, no significant differences in density were found among all the samples while, biscuits processed with fungal proteases (1.27 g/cm^3^) showed significant (*p* < 0.05) higher density with respect to those treated with the bacterial strain (1.20 g/cm^3^). The use of bacterial proteases allowed to obtain biscuits characterized with the highest specific volume (1.64 mL/g) and surface area (18155 mm^2^), whereas fungal enzymes produced biscuits with characteristics closer to those obtained using SMBS. Since gluten network is the main reason for the gas entrapment during baking, the stronger gluten matrix, the more susceptible is the structure to crack with the gas release. As a result of the higher partial proteolysis carried out by the bacterial proteases, a weaker gluten network with a better capability to entrap the gasses with less structure ruptures was formed; consequently, the specific volume and surface area of the biscuits increased while the density decreased (Sapirstein et al., 2007).

### Microstructural characteristics

SEM images of biscuit samples were captured to evaluate possible differences in their structure. Looking at Figure 3(a), (b) and (c) different microstructures can be appreciated regarding the SMBS, and protease treated biscuits. Figure 3(a) shows the structure of the biscuit containing SMBS that presented a more layered microstructure with elongated cavities and also a more aerated internal structure with very small holes. When the enzymes were used, the surface became more irregular and larger pockets appeared in the samples (Figure 3(b) and (c). The structure of the fungal protease biscuits (Figure 3(c)) presented less voids but of larger dimensions than those observed in the samples treated with bacterial enzymes (Figure 3(b)). Biscuits prepared using bacterial enzymes were characterized by a higher number of cavities of intermediate sizes, making the structure more porous. In this regard, porosity was found significantly (*p* < 0.05) higher in the samples that included bacterial proteases. The reason behind this could be the higher hydrolyzing activity of these proteases on the gluten network that would cause the porosity increase and the appearance of a greater number of voids inside the structure. This finding agreed with the results reported by López-Fandiño et al. (1991) in which *Bacillus subtilis* proteases produced more peptides with lower molecular weight from ovine and bovine caseins than those generated by *Aspergillus oryzae*.

Data from the analysis of the microstructural surface of the biscuits are reported in Table 2. The contrast value indicates the dissimilarities of the image, while homogeneity value has the opposite meaning which expresses the similarity of certain pixels of the image. Entropy value highlighted the regions in the data that are important or interesting in terms of exhibiting more variation or change in their closer areas compared with other regions, which changes in the same direction as porosity (Wang and Shen, 2011). Thus, entropy value indicates the mess in the image since the lack of repeatability in similar pixels refers to the non-uniform distribution of the image components. High values of contrast and entropy are indications of surface irregularity, in fact, the surface of the biscuits produced with bacterial protease (4b) had a rougher surface, while an opposite situation was found in the fungal enzyme treated samples (4c). On the other hand, energy value represents the brightness of image. The existence of less valleys (darker areas in the image) in an image causes higher energy based on the principles of image analysis. Therefore, as the texture porosities (dark pixels in image) increase, energy and homogeneity values show a decreasing trend (Saberi et al., 2021). These findings agreed with the characteristics observed in the SEM pictures of the samples made by a bacterial protease that presented more pockets and consequently higher values of energy and homogeneity. No significant (*p* < 0.05) differences were found between the SMBS control, and the biscuits obtained through the fungal proteases.

### Color

Data from the color evaluation of the biscuits presented in Table 2 indicate that the lightness resulted significantly (*p* < 0.05) lower in the biscuits that underwent enzymatic treatment. The observed result may be due to the non-enzymatic browning (Maillard reaction). In fact, proteolysis generated more free peptides that reacted with the reducing sugars during baking, causing a reduction in luminosity. Therefore, the higher proteolytic activity of the bacterial protease caused more reduction in L* value than the fungal one (Zamani and Abbasi, 2017). Regarding the values, proteases from the bacterial source significantly (*p* < 0.05) increased the redness (4.64) and the yellowness (16.41) and the control SMBS achieved the lowest values. Non-enzymatic browning reaction could be the reason for the increasing of a* and b* values and as a consequence, the BI value followed the same trend (Yang et al., 2021). There is a significant difference (*p* < 0.05) among the BI and ΔE of the samples; in fact, the enzymatic treatment caused an increase of these parameters with the biscuits made with bacterial proteases that obtained the highest results.

### Principal component analysis

**Figure 4.**
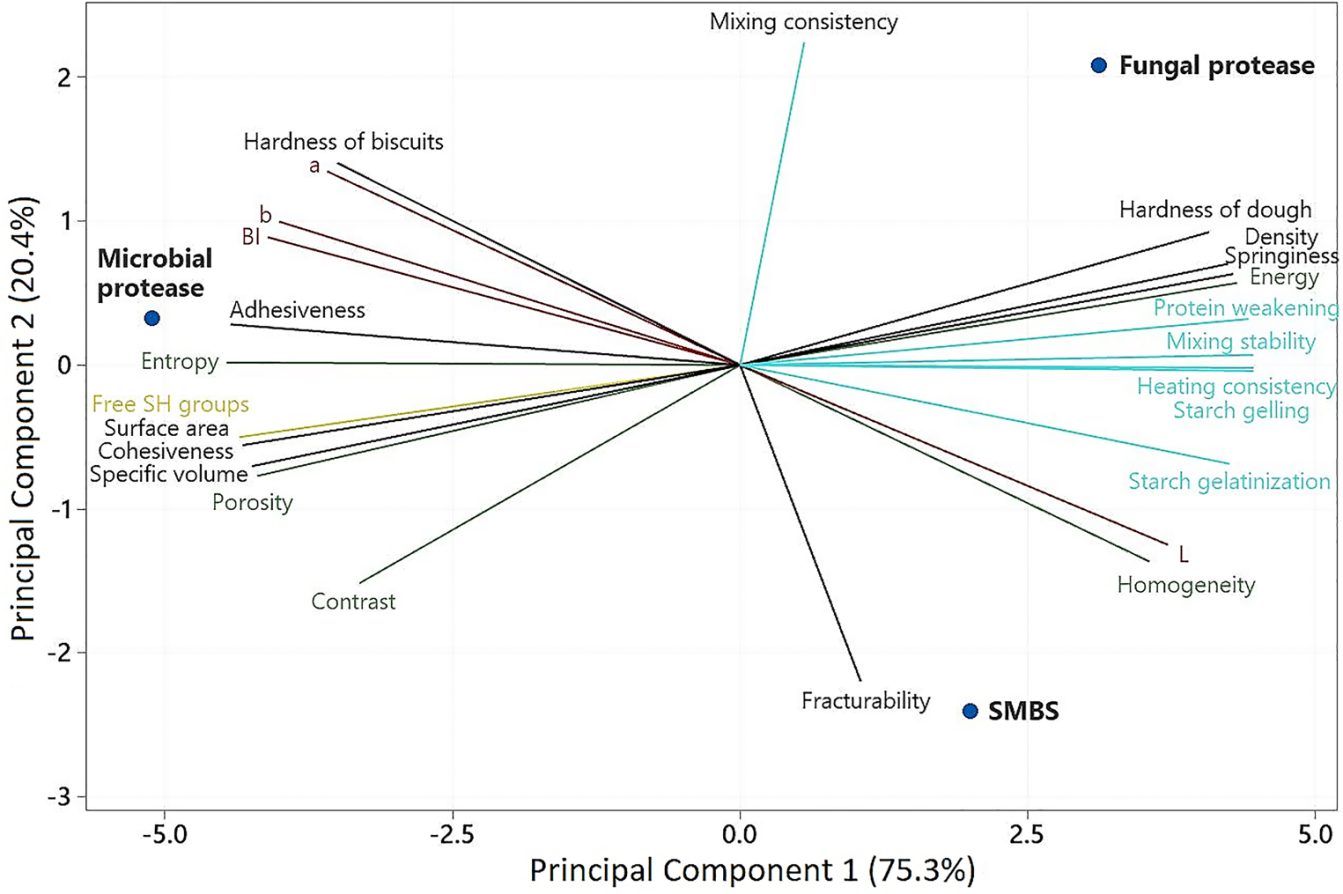
Principal component analysis of the samples treated with sodium metabisulfite and proteases.

To get a complete picture of the impact of the enzymes on the biscuits’ quality, a principal component analysis (PCA) was constructed (Figure 4), explaining 95.7% of the data variability with two components. Principal component 1 (75.3%) mainly included the variables related to the free-SH groups content, thermomechanical behavior of doughs (mixing consistency, mixing stability, protein weakening, heating consistency and starch gelling), volumetric analysis (density, specific volume and surface area) as well as dough hardness and springiness. On the other hand, factors mainly associated with the color parameters (L*, a*, b* and BI), biscuits texture (hardness and fracturability) and dough adhesiveness, belonged to the Principal Component 2 (20.4%).

The significant differences in the functionality among the SMBS and both proteases were clearly displayed in the PCA. Fungal enzyme was the protease that produces biscuits that came closest to those made with the SMBS, especially in terms of thermomechanical behavior, hardness and springiness of doughs, lightness and fracturability. In general, bacterial proteases were responsible for biscuits with higher hardness, adhesiveness and porosity.

## CONCLUSIONS

Data presented in this study described the effect of the different proteases highlighting their suitability as SMBS replacers in hard biscuit making. In terms of thermomechanical behavior, the greatest impact on the dough performance was carried out by the enzymes from the bacterial source. Samples treated with this kind of protease resulted softer than those that contained the fungal ones. On the other hand, compared with the samples containing SMBS, fungal protease slightly increased the protein weakening with a smaller reduction of the consistency during the heating stage. These outcomes were reflected in the rest of the parameters analyzed. In fact, the higher proteolytic action led by the bacterial enzymes produced a better dispersion of the hydrophobic ingredients into the proteinaceous matrix as a matter of increasing in emulsification ability of the matrix. Due to this, the enzymatically treated doughs showed higher values of cohesiveness and adhesiveness. Bacterial proteases had the greatest impact on the structure; in fact, the hard biscuits showed higher values of porosity with the spread presence of voids. Through the analysis of the principal components, it was possible to appreciate that fungal proteases were able to produce biscuits with characteristics similar to those obtained using SMBS. Outcomes confirmed that proteases from bacterial and fungal sources can represent a safer and greener alternative to using SMBS in hard biscuit making.
